# Promoting best gynecologic oncology practice: a role for the Society of Gynecologic Oncologists of Canada

**DOI:** 10.3390/curroncol13030009

**Published:** 2006-06

**Authors:** L.M. Elit, M. Johnston, M. Brouwers, M. Fung-Kee-Fung, G. Browman, I.D. Graham, on behalf of the Society of Gynecologic Oncologists of Canada, The Canadian Strategy for Cancer Control Clinical Practice Guidelines Action Group

**Affiliations:** * Department of Obstetrics and Gynecology, McMaster University, Hamilton, Ontario; † Program in Evidence Based Care, Cancer Care Ontario, Hamilton, Ontario; ‡ Department of Obstetrics and Gynecology, University of Ottawa, Ottawa, Ontario; § Department of Internal Medicine, University of Calgary, Calgary, Alberta; || School of Nursing, University of Ottawa, Ottawa, Ontario

**Keywords:** Practice guidelines, gynecologic oncology

## Abstract

During March 30–April 1, 2005, the Society of Gynecologic Oncologists of Canada (goc) and the Canadian Strategy for Cancer Control (cscc) Clinical Practice Guidelines Action Group (cpg-ag) met to

- determine how goc would like to influence practice in the care of women with gynecologic cancer.
- explore a collaborative model for developing and implementing evidence-based practice guidelines.
- investigate the utility of the cpg evaluation and adaptation cycle as a tool for selecting, adapting, and adopting guidelines.

At the workshop meeting, 21 members of the goc and the cpg-ag heard presentations from various Canadian guideline initiatives. As an example of adaptation and adoption processes, the agree (Appraisal of Guidelines for Research and Evaluation) tool was applied to guidelines in recurrent ovarian cancer, and the group explored their opportunity to use knowledge translation to influence the care of women with gynecologic cancer.

The themes influencing practice are consistent with goc’s mandate. The future is expected to involve partnering with other groups to maximize scarce resources. Resources should be directed to facilitating implementation of existing guidelines rather than to developing new documents. The full spectrum of cancer care includes prevention, screening, diagnosis, primary treatment, follow-up, treatment of recurrent disease, and palliation. High-quality evidence is available in some areas, but gaps exist where guideline panels could provide guidance. Development of a pan-Canadian gynecologic oncology process could provide an opportunity to influence access to care at the political and policy levels.

The goc will develop linkages such that the toolbox available through cscc-cpg-ag can be incorporated into future collaboration.

## 1. INTRODUCTION

Members of the Society of Gynecologic Oncologists of Canada (goc) and the Canadian Strategy for Cancer Control (cscc) Clinical Practice Guidelines Action Group (cpg-ag) came together (Table I) in March 2005 in a workshop setting. The goc is an association of physicians specializing in gynecologic cancer care, whose purpose is “to improve the care of women with gynaecologic cancer, to raise standards of practice in gynecologic oncology and to encourage ongoing research”^1^. The cpg-ag has a mandate to champion, through national collaboration, a pan-Canadian strategy to facilitate the optimal use of evidence for cancer control 2. A partnership between these two groups has the potential to address the gap between knowledge and practice in the care of women with gynecologic cancer and to reduce variation in access to best clinical care across Canada.

Clinical practice guidelines (defined as systematically developed statements meant to assist health care decisions in specific clinical circumstances) are a common mechanism for transferring evidence from clinical research into practice and for influencing clinician behaviour. Several provinces develop practice guidelines, and additional guidelines are available from other groups within and outside of Canada. Unfortunately, existing guidelines that address a given clinical situation may vary with regard to the methodologic quality of their development and to the recommendations made. These inconsistencies can cause confusion for clinicians and patients. Additional challenges can arise from suboptimal dissemination and implementation strategies. Guidelines are meant to enhance care, and the barriers that hamper the translation of new knowledge and guideline recommendations into clinical practice can also hinder effective uptake of other types of systematic clinical advice, such as targeted messaging and clinical leadership.

Not all institutions, provinces, and professional associations have the resources necessary to develop, disseminate, implement, and evaluate guidelines. And in any case, reviewing a common evidence base and developing parallel guidelines represents a duplication of effort that does not make sense in the face of numerous competing demands on scarce resources. Where high-quality guidelines exist, adoption—with or without adaptation of the recommendations to reflect local circumstances and values—may be preferable to developing new guidelines. Canada has people with acknowledged expertise in both guideline development and guideline adaptation; many of those experts participated in the workshop.

Groups such as goc not only can add credibility to guidelines by endorsing them, but they can also provide vital networks for guideline dissemination, implementation, and evaluation. As education influentials in the gynecologic oncology community, goc members can also make a unique contribution to interpretation of evidence, provision of clinical context, identification of gaps in the evidence, and provision of well-reasoned expert opinion to fill the gaps until further research is completed.

With those issues in mind, the workshop agenda focused on three sets of objectives:

- To determine if goc wants to actively influence practice in the care of women with gynecologic cancer, and if so, the methods to be used. Specifically, to determine if goc should take an active leadership role in guideline development, adaptation, implementation, or evaluation, and to determine the extent to which such activities are to be evidence-based.
- To explore a collaborative model for designing a pan-Canadian strategy to develop and implement evidence-based clinical practice guidelines for gynecologic oncology.
- To investigate the utility of an established methodology, the cpg Evaluation and Adaptation Cycle 3, as a tool for selecting, adapting, and adopting guidelines for gynecologic oncology—starting with a pilot test of the Adaptation Cycle focusing on the management of recurrent ovarian cancer. The Cycle would be used to generate specific recommendations for the Canadian context.

The present report describes discussions related to the first two sets of objectives. Methods, results, and conclusions associated with the cpg Evaluation and Adaptation Cycle will be described in a separate report.

## 2. MATERIALS AND METHODS

As a preliminary step for all workshop activities, participants were provided with common background information through a review of three different provincial approaches (those of British Columbia, Ontario, and Quebec) for developing practice guidelines and treatment policies, a summary of the experience of the Society of Obstetricians and Gynecologists of Canada (sogc) with national guidelines, and an overview of cpg adaptation and adoption models and methods 3–5. A specific example of a contentious clinical problem—treatment of recurrent ovarian cancer—was explored through a review of the current clinical issues, appraisal by workshop participants of three available guidelines (from the Cancer Care Ontario Program in Evidence-based Care 6, the Scottish International Guidelines Network 7,8, and the British Columbia Cancer Agency 9), and a comparison of the recommendations made in the three guidelines plus supporting information in the form of two other advice documents (from the National Cancer Institute Physician Data Query Web site 10 and the National Institute for Health and Clinical Excellence 11,12).

Following those activities, breakout groups (each consisting of six people) addressed all three key questions related to goc’s role in influencing practice:

- Does goc want to influence practice in women with gynecologic cancer?
- What methods should goc use to inform practice at a pan-Canadian level?
- What role should an evidence-based process play in influencing practice?

Following small-group discussion of each question, the individual groups reported their thoughts for further discussion by all participants before moving to the next question.

## 3. RESULTS

Issues emerging from discussion during the breakout and plenary sessions related to four key themes:

- The current and future role of goc
- Making the most of limited resources
- Providing guidance across the full continuum of care
- Influencing funding for cancer care with a view to facilitating equity of access across provinces

Workshop participants agreed that influencing practice is consistent with goc’s mandate and is supported by its mission statement. Moreover, goc may be the group most likely to effectively influence practice across Canada. Currently, goc is viewed as having an advisory and educational role. The group could build on that experience by developing a more “outward looking” approach that would move their influence beyond goc’s membership to other specialty groups and trainees and that would solidify goc’s role as a knowledge broker for other clinicians. This process could start by dealing with the large unfilled demand from gynecologists outside cancer centres for leadership and high-quality, implementable guidelines related to cancer care.

Some debate occurred about the range of guideline topics that goc should consider, leading to the conclusion that oncology topics (such as systemic therapy for ovarian cancer) should be tackled first. Strengths on which goc can draw include its interdisciplinary approach and its experience with continuing medical education over the Internet.

In addition to aiding in clinical decision-making, practice guidelines can have educational value. Plans to endorse information for patients with gynecologic cancer could also be incorporated into a program for influencing practice. In mapping out future activities, goc should consider developing partnerships with other groups to maximize scarce resources and to explore funding opportunities. One of these partners might be the sogc, but the relationship between goc and the sogc with respect to guideline development and dissemination would need to be clearly defined. For example, goc guidelines would likely be developed independently of sogc, but sogc members could provide feedback on draft guidelines, and the sogc may wish to endorse goc guidelines for use by gynecologists across Canada.

The number of oncologists across Canada is limited. To address the need for additional resources, the health care system should engage and educate a broad range of practitioners (obstetrician–gynecologists, family physicians, etc.) to provide some aspects of care to women with gynecologic cancers.

The human resource issues for providing care are also reflected in the limited number of individuals available to develop guidelines and other mechanisms to influence practice. In many cases, the same clinicians who deliver care to patients and act as practice leaders would be the key players in guideline development. In light of limited infrastructure and resources for influencing practice, workshop participants wondered if such resources should be directed to facilitating implementation of existing guidance rather than to developing new guidance documents. Broad participation in guideline- and policy-development activities may require a cultural shift for some practice communities so that time is allocated for this work. Such a shift may be more challenging in some settings (the surgical community, for example) than in others. However, successful involvement of clinicians across Canada in guideline development, endorsement, dissemination, and implementation has the potential to build capacity, reduce duplication of effort, and improve patient care and outcomes. Evaluating the impact of these new activities on practice and patient outcomes will be critical.

The full spectrum of cancer care includes prevention, screening, diagnosis, primary treatment (surgery, radiotherapy, chemotherapy), follow-up, treatment of recurrent disease, and palliation. The availability of high-quality evidence varies across this continuum, as does the need for guidance to clinicians and patients. In some cases, guidance is needed in circumstances where evidence of effectiveness is absent, inconsistent, or unclear. The result can be clinical uncertainty and variation in practice. Evidence-based guidelines help to inform clinical decisions by making recommendations based on a systematic review and interpretation of the available evidence. Where gaps in the evidence exist, guideline panels could provide guidance by using a formalized process to make recommendations based on expert opinion.

Activities other than practice guidelines that may influence care include continuous professional development (cpd), patient advocacy, lobbying, treatment policies, algorithms, care paths, consensus statements, patient information, and clinical research. Many of these activities can be informed by evidence-based practice guidelines.

Developing a pan-Canadian perspective on key issues in gynecologic oncology could provide the opportunity to influence funding and access to care at the political and policy level. It could also allow clinicians to organize themselves as advisors and knowledge brokers to funding agencies. To effectively engage in such activities—and in others suggested at the workshop—collaboration across provincial boundaries would be necessary.

## 4. DISCUSSION

The goc wants to use partnerships with other groups such as the sogc, the Canadian Association of Radiation Oncologists, the Canadian Association of Medical Oncologists, the National Ovarian Cancer Association, provincial specialty groups, guideline developers in the provinces, and the cscc-cpg-ag to influence practice in women with gynecologic cancer. The methods that goc should use to inform practice include identifying unmet needs for clinical advice in managing gynecologic cancers—for example, defining and developing a broad but relevant audience, and exploring opportunities to build accountability among clinicians for participating in guideline-related activities. The Society can build on work already being done for cpd. The goc Web site, with access through the sogc Web site, could be developed as a communication tool for these and future activities.

For the time being, efforts should concentrate on guideline implementation issues, because developing practice guidelines requires infrastructure and resources not currently available to goc. However, adoption or adaptation of guidelines developed by others may be possible. At the workshop, goc members expressed interest in participating in research on the further development of guideline adaptation processes. They also suggested using goc opinion leaders to build consensus among practitioners. The evidence-based process (including guideline adaptation) was endorsed as an approach to providing advice to clinicians.

The Society is in an ideal position to identify common issues in practice as priorities for guidance documents across the entire spectrum of care in gynecologic oncology, from prevention through to palliation. The Society’s role is to promote a culture of evidence-based practice in gynecologic oncology. As clinical experts, goc members could also respond to emerging evidence.

In addition to endorsing or adapting evidence-based guidelines from other groups, goc could consider partnering with groups that develop evidence-based guidelines to work on new guidelines or to update existing guidelines. Such work should be based on a defined process or structure, with involvement of appropriate experts in guideline and clinical research methods. Criteria—for example, need, potential impact on care, quality of the evidence base, availability of an existing systematic review or guideline—should be defined for choosing topics for guideline development or adaptation. Guidelines and other advice documents should include the patient perspective and an economic evaluation. When available, existing systematic reviews and good-quality guidelines could be the starting point for an evidence-based approach. Where evidence is insufficient, a formalized consensus process could be used to develop advice.

The Society could also contribute by identifying gaps in the evidence and advocating for appropriate clinical trials to fill those gaps. With its partners, goc should develop explicit implementation and evaluation strategies based on evidence of effectiveness.

## 5. CONCLUSION

Overall, participants concurred that the workshop was a positive experience. Based on the presentations and ensuing discussion, they concluded that effective tools exist for guideline development, adaptation, and adoption, but that delivering guidelines to practitioners during the patient encounter presents a significant challenge. It became clear during the workshop that goc and cpg-ag have common objectives with respect to influencing clinical practice.

When the workshop took place, the ag was still determining its mandate, the scope of its activities, and a model for a pan-Canadian approach. Representatives of the ag found the workshop to be useful as a pilot test for developing beneficial partnerships in the future. The goc representatives concluded that goc has a mandate to use clinical education to influence care and that this mandate may extend to other activities such as providing evidence-based advice to practitioners.

## Figures and Tables

**Table I:** Workshop participants

Members of the Society of Gynecologic Oncologists of Canada (goc)
Peter Craighead, Alberta
Lesa Dawson, Newfoundland
Laurie Elit, Ontario
Michael Fung Kee Fung, Ontario
Prafull Ghatage, Alberta
Walter Gotlieb, Quebec
Robert Lotocki, Manitoba
Dianne Miller, British Columbia
Joan Murphy, Ontario
Diane Provencher, Quebec (goc President)
Barry Rosen, Ontario
Rejean Savoie, New Brunswick
Luis Souhami, Quebec
Gavin Stuart, British Columbia
Ken Swenerton, British Columbia
Members of the Canadian Strategy for Cancer Control Clinical
Practice Guidelines Action Group (cscc-cpg-ag)
Melissa Brouwers, Ontario
George Browman, Alberta (cscc-cpg-ag Chair)
Ian Graham, Ontario
Louise Paquet, Quebec
Jill Petrella, Nova Scotia
Coordinator of the Clinical Practice Guidelines Adaptation
Project
Louise Zitzelsberger
